# Editorial: Evolving roles of piRNAs in solid tumors

**DOI:** 10.3389/fonc.2023.1178634

**Published:** 2023-04-11

**Authors:** Lui Ng, Alfons Navarro, Wai-Lun Law

**Affiliations:** ^1^ Department of Surgery, School of Clinical Medicine, Li Ka Shing Faculty of Medicine, The University of Hong Kong, Hong Kong, Hong Kong SAR, China; ^2^ Faculty of Medicine, University of Barcelona, Barcelona, Spain

**Keywords:** piRNA, PIWI, biomarker, non-coding RNA, solid tumor

According to Global Cancer Statistics 2020, an estimated 19.3 million new cancer cases and almost 10 million cancer deaths occurred in 2020 (1). Solid tumors represent approximately 90% of adult human cancers, hence they warrant significant attention from the research fraternity to improve upon the existing platforms of treatment and management of the malignancy. Only by a better understanding of the biology associated with cancer development and progression can we identify clinically relevant novel molecular targets that can not only improve upon the risk stratification of the patients, but also assist in overall disease management.

PIWI-interacting RNA (piRNA) is a class of small non-coding RNA (26-31nt) that interacts with PIWI proteins to form the piRNA silencing complex (piRISC). PIWI is a subfamily of Argonaute, and piRNA must bind to PIWI to exert its regulatory role, although have been also described PIWI independent functions for piRNAs. Nearly 10 million unique piRNA sequences (2) have now been identified in humans alone that have been recognized to play a wide variety of roles including germline development, maintenance, and protection of the genome integrity by repressing the activity of transposons through post-transcriptional silencing or other epigenetic mechanisms. Emerging data suggests that piRNAs also have strong regulatory roles within the somatic tissues where they regulate gene expression by inducing histone modification and DNA methylation. Owing to their remarkable roles in maintaining cellular homeostasis, it is not surprising that the expression of piRNAs is reported to be frequently deregulated in several cancers. Current studies indicated that piRNAs are significantly abnormally expressed and are involved in the initiation, progression, and metastasis of different solid tumors, which may be the potential diagnostic tools, prognostic markers, and therapeutic targets for cancers. This special issue is a collection of original research and review articles on this topic.

In the first article included, Liu et al. performed a piRNA profiling in non-small cell lung cancer (NSCLC) adenocarcinoma patients. Comparing the expression between 20 tumor and its paired normal tissue they identified 1333 downregulated and 236 upregulated piRNAs. Then, the authors focused on the role of piR-211106, which was significantly downregulated in tumor tissue and in 2 lung cancer cell lines (A549 and HCC2279). Overexpression and knockdown studies showed that piR-211106 was acting as a tumor suppressor gene, regulating proliferation, apoptosis, and migration. Additionally, piR-211106 mediated cisplatin resistance. To identify which genes could be regulated by this piRNA, the authors performed RNA pull down experiments, which allowed the identification of several downstream targets, including pyruvate carboxylase (*PC*), a metabolism enzyme that has been previously found overexpressed in NSCLC. Overexpression of piR-211106 reduced *PC* levels, mRNA and protein. They argued that according to the expression of piR-211106 in both cytoplasm and nucleus (according to FISH data) it could work in two ways: triggering *PC* expression in the nucleus by methylation regulation; and/or regulating PC levels on cytoplasm. Both mechanisms end in tumor growth suppression, which underlines that piR-211106 is a new therapeutic and prognostic biomarker in NSCLC.

Cui et al. analyzed the role of the opioid agonist Butorphanol, which traditionally is used for postoperative analgesia, as a potential therapeutic agent for osteosarcoma. The authors evidenced that Butorphanol treatment in 2 osteosarcoma cell lines (MG63 and U2OS) was able to inhibit cell proliferation and migration. Interestingly, the authors concentrated on the study of the role of piRNAs in this process, identifying several piRNAs which expression was modulated by the opioid. PiRNA sequencing in MG63 cell line treated with butorphanol, identified 8 differentially expressed piRNAs, including piR_006613. PiR_006613 overexpression *in vitro* inhibited the proliferation and migration of both cell lines, while the inhibition produced the inverse effect. Moreover, the effect previously observed with the single administration of butorphanol, was reduced when piR_006613 was silenced, indicating a dependent effect. *FN1* was identified and validated by the authors as a target of piR_006613. PiR_006613 through targeting *FN1* translation was involved in the antitumorigenic role of Butorphanol in osteosarcoma.

In Yao et al. review, the authors summarized some piRNAs aberrantly expressed in various solid cancers that were shown to regulate cancer cell properties including proliferation, invasion/migration, cell cycle progression and chemoresistance. The underlying molecular mechanism, such as effects on epithelial–mesenchymal transition (EMT) pathway and epigenetic modification, were discussed. Notably, some of these piRNAs could be potential therapeutic approach to treat cancer since they were able to induce cell cycle arrest or enhance sensitivity of cancer cells to chemotherapy. In addition to the functional roles of piRNA in cancer, Yao et al. suggested that single-nucleotide polymorphisms (SNPs) of certain piRNAs or PIWI were potential biomarkers for tumor risk assessment, which was supported by findings from several studies regarding SNPs of certain piRNAs or PIWI were associated with risk of cancer development or disease-free survival of patients. Finally, the authors summarized some piRNAs or PIWI which are potential diagnostic or prognostic biomarkers for cancer patients.

In Jian et al. mini-review, they discussed the new findings of piRNAs specifically in lung cancer, including their biosynthetic processes, mechanisms of gene suppression, as well as the significance of these piRNAs tested in lung cancer samples to determine their involvement in cancer progression. They summarized the findings of 13 piRNAs aberrantly expressed in lung cancer, such as their pattern of dysregulation, functional effects, molecular mechanism involved and their potential clinical application. Finally, the authors suggested some critical obstacles that researchers should notice during investigation of piRNAs or piRNA/PIWI complex.

Since the first discovery of piRNA in 2006, there have been increasing number of studies investigating this new class of small non-coding RNA. Though this research field is still in its infancy, we are gaining deeper understanding on the biogenesis of piRNA and the roles of piRNAs in cancers. We hope this special issue can inspire and ignite further research on piRNAs and pave the way to their clinical application in cancer patients in the near future.

## Author contributions

LN and AN wrote the Editorial. W-LL reviewed the Editorial. All authors contributed to the article and approved the submitted version.
